# EEG-Derived Entropy Monitoring During Propofol Sedation for ERCP: Sedation Profiles, Age-Related Effects, and Implications for Procedure-Specific Target Ranges

**DOI:** 10.3390/medicina62061047

**Published:** 2026-05-28

**Authors:** Sonia Elena Popovici, Stelian Adrian Ritiu, Ioan Sporea, Dorel Sandesc, Ovidiu Horea Bedreag, Marius Păpurică, Alina Popescu

**Affiliations:** 1 Faculty of Medicine, Victor Babes University of Medicine and Pharmacy, 300041 Timisoara, Romania; popovici.sonia@umft.ro (S.E.P.); popescu.alina@umft.ro (A.P.); 2 Doctoral School, Victor Babes University of Medicine and Pharmacy, Eftimie Murgu Square 2, 300041 Timisoara, Romania; 3 Anaesthesia and Intensive Care Research Center (CCATITM), Victor Babes University of Medicine and Pharmacy, 300041 Timisoara, Romania; 4 Advanced Regional Research Center in Gastroenterology and Hepatology, Department VII: Internal Medicine II, Discipline of Gastroenterology and Hepatology, Victor Babes University of Medicine and Pharmacy, 300041 Timisoara, Romania; isporea@umft.ro; 5 Clinic of Anaesthesia and Intensive Care, Emergency County Hospital “Pius Brînzeu”, 300723 Timisoara, Romania

**Keywords:** ERCP, gastrointestinal endoscopy, propofol sedation, EEG-derived monitoring, State Entropy, procedural sedation, depth of anesthesia, procedure-specific sedation targets

## Abstract

*Background and Objectives*: Conventional EEG-derived sedation targets for Entropy monitoring (State Entropy SE 40–60) were established in the context of general anaesthesia and may not be directly applicable to procedural sedation for endoscopic retrograde cholangiopancreatography (ERCP). This study aimed to characterize SE and Response Entropy (RE) trajectories during propofol-based sedation for ERCP and to evaluate their relationships with clinical sedation depth, patient characteristics, and procedural outcomes. *Materials and Methods:* In this prospective, single-center observational study, 50 consecutive adult patients undergoing elective ERCP under propofol-based sedation were enrolled. SE and RE were recorded at baseline and at serial intra-procedural timepoints. Time-in-zone analysis classified SE values into predefined ranges. Correlations between Entropy indices and MOAA/S scores, patient factors, Aldrete recovery scores, and adverse events were assessed using Spearman’s rank correlation. *Results*: The median patient age was 72.5 years (IQR 65.0–79.0), and the median ASA score was 3 (IQR 2–3). Following induction, SE declined from a baseline mean of 89.3 ± 1.5 to a mean of 68.8 ± 5.3 at 10 min, the lowest group-level value recorded; the mean individual SE nadir across patients was 67.2 ± 5.3. No SE values below 40 were observed at any timepoint. Mean time spent within the conventional SE 40–60 target range was 3.7% ± 10.6, while mean time within SE 60–85 was 80.7% ± 8.3. SE at 3 min correlated moderately with MOAA/S at 3 min (Spearman rho = 0.430, *p* = 0.002), with substantial within-category variability. Age showed a strong negative correlation with SE at 3 min (rho = −0.612, *p* < 0.001), an effect that persisted at 5 min, consistent with deeper early EEG suppression in older patients, which may reflect increased pharmacodynamic sensitivity, age-related changes in spectral substrate, or both. ASA score was associated with SE at 15 min only (rho = −0.299, *p* = 0.035). Patients who experienced adverse events demonstrated higher SE instability, though differences did not reach statistical significance. Recovery was rapid, with a median Aldrete score of 10 at 15 min. *Conclusions*: During propofol sedation for ERCP, observed SE values operated predominantly between 60 and 85, well above the conventional general anaesthesia target of 40–60. Older age was the strongest predictor of early sedation depth. These findings suggest that in elderly, high-ASA patients receiving propofol-based multi-drug sedation for ERCP, numerical SE values differ systematically from conventional general anaesthesia-derived target ranges. Whether this reflects true procedure-specific sedation requirements or cohort-specific spectral substrate differences warrants prospective outcome-anchored investigation.

## 1. Introduction

Endoscopic retrograde cholangiopancreatography (ERCP) is a highly stimulating endoscopic procedure and commonly requires deep propofol-based sedation to ensure procedural conditions and patient tolerance. At the same time, sedation for ERCP is characterized by a narrow therapeutic window, where insufficient depth may compromise procedural success and excessive depth may increase the risk of respiratory and hemodynamic adverse events [1].

Sedation depth in routine practice is usually assessed using intermittent clinical scales such as the Modified Observer’s Assessment of Alertness/Sedation (MOAA/S). While clinically useful, these scales are inherently discontinuous and offer limited granularity once patients become poorly responsive [2,3]. Electroencephalography-derived monitoring methods, including Entropy, provide a continuous measure of hypnotic depth and may capture variability not detected by clinical assessment alone. Previous studies have demonstrated that EEG-derived indices such as bispectral index (BIS) and Entropy correlate with clinical sedation scales, while providing additional resolution during procedural sedation [4].

However, the interpretation of Entropy values during procedural sedation remains uncertain. Conventional target ranges for EEG-derived indices were established in the context of general anaesthesia, where values between 40 and 60 are considered indicative of adequate hypnosis. These targets may not translate directly to endoscopic sedation, where procedural stimulation and lighter intended planes of sedation alter the clinical context. Prior studies in gastrointestinal endoscopy have suggested that clinically adequate sedation often occurs at higher EEG-derived index values than those associated with general anaesthesia [5,6].

BIS monitoring during gastrointestinal endoscopy has been investigated in several studies, but data specific to Entropy-derived indices—State Entropy (SE) and Response Entropy (RE)—in the context of ERCP are limited. BIS and Entropy are algorithmically distinct: whereas BIS is derived from a proprietary weighted composite of EEG features, Entropy quantifies the spectral irregularity of the EEG and frontalis EMG signals, yielding SE and RE as separate outputs. These algorithmic differences mean that BIS-derived numerical target ranges cannot be directly transferred to Entropy monitoring. Nevertheless, BIS studies in gastrointestinal endoscopy provide relevant contextual information regarding the expected direction of EEG-derived index values during procedural sedation, even if precise numerical thresholds are not interchangeable. Furthermore, prior studies of EEG-derived monitoring during endoscopy have predominantly enrolled patients undergoing colonoscopy or upper endoscopy—populations that differ meaningfully from ERCP patients in terms of age, comorbidity burden, procedural duration, and stimulation profile. Whether the conventional SE 40–60 target range, derived from general anaesthesia, appropriately characterizes sedation depth during ERCP remains unknown.

While Entropy monitoring has been used as a titration tool during ERCP sedation, no study has systematically characterised SE and RE temporal trajectories during propofol-based ERCP sedation or evaluated whether the conventional SE 40–60 target range—established in general anaesthesia—accurately reflects sedation depth in this specific procedural context. This gap is clinically relevant given the distinct patient population, procedural stimulation profile, and intended sedation plane associated with ERCP.

The present study aimed to characterize SE and RE trajectories during propofol sedation for ERCP, to evaluate their relationship with MOAA/S, and to explore associations with patient characteristics, recovery, and adverse events.

## 2. Materials and Methods

### 2.1. Study Design and Setting

This prospective, single-center observational study was conducted at the “Pius Brînzeu” Emergency County Hospital, Timișoara, Romania, between November 2025 and January 2026. All enrolled patients received continuous Entropy monitoring using State Entropy (SE) and Response Entropy (RE), in addition to standard monitoring, throughout the procedure. The study was performed in accordance with the Declaration of Helsinki and was approved by the Institutional Review Board of the “Pius Brînzeu” Emergency County Clinical Hospital Timișoara (approval number 569/8 October 2025) and the Institutional Ethics Committee for Scientific Research of the “Victor Babeș” University of Medicine and Pharmacy Timișoara (approval number 15/2022, revision 2026). Written informed consent was obtained from all participants.

This observational study was not prospectively registered, as registration is not mandated for observational studies under applicable institutional guidelines.

### 2.2. Patient Selection

Adult patients scheduled for elective ERCP with planned propofol-based procedural sedation were consecutively assessed for eligibility.

Inclusion Criteria: age ≥ 18 years, elective ERCP under propofol-based sedation administered by an anesthesiologist, American Society of Anesthesiologists (ASA) physical status I–III, and written informed consent.

**Figure 1:**
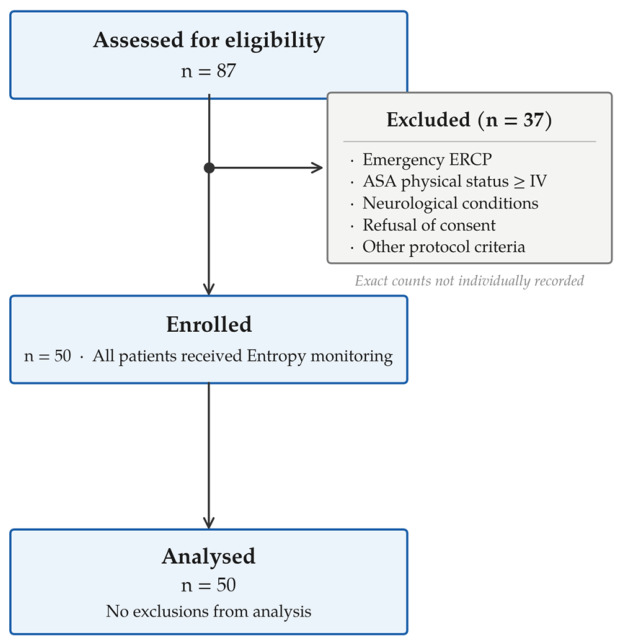
Patient flow diagram. Of 87 patients assessed for eligibility, 37 were excluded based on prespecified criteria and 50 were enrolled. All enrolled patients received continuous Entropy monitoring and were included in the final analysis.

Exclusion Criteria: known hypersensitivity to study medications, neurological conditions or treatments interfering with EEG monitoring (e.g., seizure disorder, recent stroke, severe dementia, chronic sedatives), emergency ERCP, planned general anesthesia with endotracheal intubation, ASA ≥ IV, pregnancy or lactation, refusal or inability to provide consent, and inadequate Entropy signal acquisition. The patient flow is illustrated in Figure 1.

### 2.3. Sedation Protocol and Procedural Management

Propofol-based sedation was delivered by anesthesiologists, with dosing adjusted according to routine clinical practice in order to balance procedural requirements and cardiorespiratory stability. All procedures were performed in a dedicated ERCP suite equipped with standard monitoring, resuscitation equipment, and an anesthesia machine. Upon arrival, peripheral intravenous access was established and balanced crystalloid infusion was initiated, while supplemental oxygen at a flow rate of 2–4 L/min was administered via nasal cannula throughout the procedure. Patients were positioned in the left lateral semi-prone position.

Propofol (Fresenius Kabi, Bad Homburg, Germany) was administered as an initial bolus of 0.5 mg/kg, followed by intermittent boluses at the discretion of the attending anesthesiologist. Fentanyl (Fentanyl Kalceks, 50 mcg/mL,Akciju, Laboratoire Aguettant, Lyon, France) was administered as a premedication at a dose of 50 μg, with additional intra-procedural boluses given when clinically indicated, while midazolam (Midazolam Hypericum, 5 mg/mL, Laboratoire Aguettant, Lyon, Franc) at doses of 1–2 mg was administered at the discretion of the attending anesthesiologist when additional anxiolysis was considered appropriate. Additional adjunctive medications were administered as required based on clinical judgment. The target level of sedation was moderate to deep sedation, sufficient to ensure patient immobility and optimal procedural conditions while maintaining cardiorespiratory stability. All ERCP procedures were performed by one of three senior endoscopists with extensive experience in advanced endoscopic procedures.

### 2.4. Monitoring and Sedation Assessment

Standard monitoring included continuous electrocardiography, non-invasive blood pressure measurement, and pulse oximetry. Sedation depth was assessed clinically using the Modified Observer’s Assessment of Alertness/Sedation (MOAA/S) scale and was continuously monitored using State Entropy (SE) and Response Entropy (RE) obtained from a CARESCAPE B650 monitor (GE Healthcare, Helsinki, Finland). Entropy values were recorded for study purposes and did not directly guide sedation titration. Endoscopists were not provided access to entropy monitor displays and were therefore blinded to entropy values throughout the procedure. Sedation titration remained at the discretion of the attending anaesthesiologist based on clinical assessment. Clinical sedation assessment using the MOAA/S score remained the primary reference for sedation depth in routine practice. Entropy sensors were applied prior to any drug administration, and baseline values were recorded while patients were fully awake in order to verify signal acquisition.

The MOAA/S score was assessed at predefined timepoints concurrent with Entropy recordings, namely at baseline prior to sedation and at 3, 5, 10, 15, 20, and 25 min after induction. The targeted sedation depth corresponded clinically to a MOAA/S score of 2 to 3.

### 2.5. Outcomes

The primary outcome of the study was the intra-procedural profile of State Entropy, including its temporal evolution and the distribution of values across predefined sedation ranges using a time-in-zone approach.

Secondary outcomes included the relationship between SE values and clinical sedation depth as assessed by the MOAA/S score, the association between Entropy values and patient characteristics such as age and ASA physical status, the relationship between intra-procedural Entropy measures and post-procedural recovery assessed using the Aldrete score at discharge from the recovery area, and the association between within-patient SE variability and the occurrence of adverse events, including hypotension and desaturation.

### 2.6. Definitions

State Entropy nadir was defined as the lowest SE value recorded during the procedure, while SE variability was defined as the range of SE values observed within each patient, calculated as the difference between maximum and minimum values. For the time-in-zone analysis, SE values were classified into predefined ranges corresponding to deep sedation for values below 40, general anesthesia range for values between 40 and 60, light to moderate sedation for values between 60 and 85, and minimal sedation for values above 85. The proportion of time spent within each range was calculated for each patient based on discrete recorded timepoints, without interpolation between measurements.

Hypotension was defined as a systolic blood pressure below 90 mmHg or a decrease of at least 20% from baseline values, desaturation as a peripheral oxygen saturation below 90%, and bradycardia as a heart rate below 50 beats per minute. A composite anesthetic adverse event outcome was defined as the occurrence of any of these events during the procedure.

### 2.7. Statistical Analysis

Statistical analyses were performed using Python version 3.12 with the pandas 2.2, scipy 1.13, statsmodels 0.14, and scikit-learn 1.5 libraries. The normality of continuous variables was assessed using the Shapiro–Wilk test. Continuous variables are presented as median and interquartile range, while categorical variables are reported as counts and percentages. Group comparisons were performed using the Mann–Whitney U test or the Kruskal–Wallis test for continuous variables and the chi-square test or Fisher’s exact test for categorical variables, as appropriate. Correlations between variables were assessed using Spearman’s rank correlation coefficient. Between-group differences in SE instability were evaluated using the Mann–Whitney U test. A two-sided *p*-value below 0.05 was considered statistically significant.

No a priori sample size calculation was performed, as the primary outcome was descriptive. With a sample size of 50 patients and observed SE standard deviations ranging between 4.4 and 7.4 across procedural timepoints, the 95% confidence interval around each group-level mean SE estimate was approximately ±1.5 to 2.0 points, providing sufficient precision to characterize trajectory profiles relative to the conventional SE target range. For secondary correlation analyses, this sample size provides approximately 80% statistical power to detect Spearman correlation coefficients of at least 0.38 at a two-sided alpha level of 0.05.

No correction for multiple comparisons was applied, as all correlation analyses were pre-specified as exploratory. Given the number of correlations tested across repeated timepoints, the possibility of type I error cannot be excluded, and statistically significant findings should be interpreted accordingly.

The number of patients contributing data decreases at later timepoints, reflecting procedure completion: all 50 patients contributed data at 3, 5, 10, and 15 min, while 35 patients contributed at 20 min and 15 at 25 min. Patients with shorter procedures completed and exited monitoring before these timepoints.

During the preparation of this manuscript, artificial intelligence tools were used to assist with language editing and code development. All outputs were critically reviewed by the authors, and all scientific content remains the responsibility of the authors.

## 3. Results

### 3.1. Patient Characteristics

During the study period, 87 patients were assessed for eligibility, of whom 50 met the inclusion criteria and were enrolled. No patients were excluded due to failed Entropy signal acquisition. The median age was 72.5 years (IQR 65.0–79.0), and 24 patients (48.0%) were male. Most patients were classified as ASA physical status II–III, with a median ASA score of 3.0 (IQR 2.0–3.0). Hypertension was present in 62.0% of patients. Antiplatelet therapy and statin use were reported in 36.0% and 40.0% of patients, respectively. Procedural duration was 22.5 min (IQR 20.0–30.0), and stent placement was performed in 58.0% of cases, with malignant diagnoses present in 56.0% of the subjects.

The median propofol dose was 130.0 mg (IQR 105.0–185.0). Sedation-related adverse events occurred in a proportion of patients consistent with published rates for propofol-based ERCP sedation, including hypotension in 16.0% and desaturation in 12.0%. Despite this, recovery was rapid, with a median Aldrete score of 10.0 (IQR 9.0–10.0) at 15 min, indicating that adverse events were transient and did not substantially delay recovery. Full baseline characteristics are presented in Table 1.

### 3.2. Intra-Procedural Entropy Trajectories

**Figure 2:**
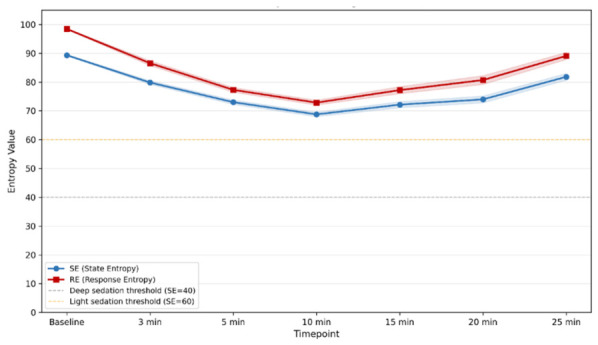
Trajectories of State Entropy (SE) and Response Entropy (RE) during ERCP. Mean SE and RE values are shown at baseline and at predefined intra-procedural timepoints. Both indices declined following induction, reaching a nadir at 10 min, and subsequently increased during recovery. RE values remained consistently higher than SE at all timepoints. The dashed lines indicate the threshold for deep sedation (SE = 40) and the lower bound of the conventional general anaesthesia target range (SE = 60). Shaded areas represent ± one standard deviation around the mean at each timepoint.

State Entropy (SE) and Response Entropy (RE) trajectories across the study group are presented in Table 2 and illustrated in Figure 2. Baseline values, prior to any drug administration, were tightly clustered, with SE median at 90.0 (IQR 88.0 to 90.8, mean 89.3 ± 1.5) and RE at 98.5 (IQR 98.0 to 99.8, mean 98.4 ± 1.3), indicating a uniform awake starting point across the cohort and intact frontal neuromuscular activity in all patients at the start of monitoring.

Following propofol induction, both indices declined rapidly. At 3 min, mean SE decreased to 79.8 ± 4.4 (median 80.0, IQR 78.0 to 82.0), corresponding to a reduction of approximately 9.5 points from baseline. SE continued to decline to 73.0 ± 5.2 at 5 min (median 74.0) and reached its lowest mean value at 10 min (68.8 ± 5.3). Thereafter, SE progressively increased, reaching 72.1 ± 7.4 at 15 min, 74.0 ± 6.9 at 20 min, and 81.8 ± 4.5 at 25 min.

### 3.3. Relationship Between State Entropy and Clinical Sedation (MOAA/S)

**Figure 3:**
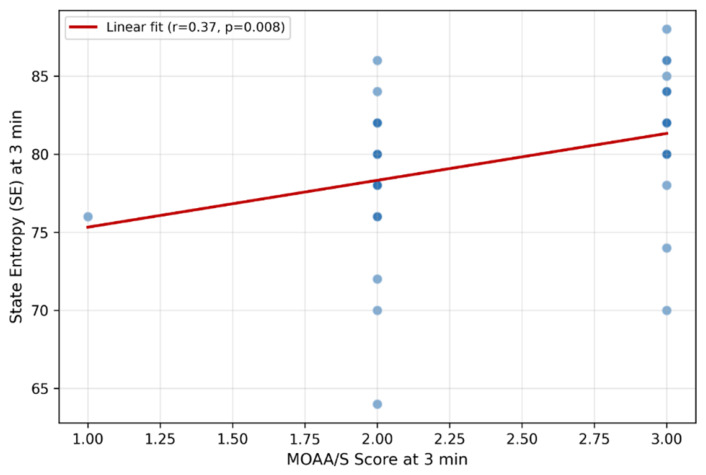
Relationship between State Entropy (SE) and MOAA/S score at 3 min. Scatter plot showing the association between SE values and clinical sedation depth assessed by the Modified Observer’s Assessment of Alertness/Sedation (MOAA/S) score at 3 min. MOAA/S scores at 3 min ranged from 1 to 3 in this cohort. The red line represents the linear fit (Pearson r = 0.37, *p* = 0.008).

The relationship between SE and MOAA/S was examined at 3 min, the earliest post-induction timepoint at which MOAA/S was recorded, to assess the convergent validity between objective EEG-derived depth and clinical behavioral assessment. SE showed a moderate positive correlation with MOAA/S at 3 min (Spearman rho = 0.430, *p* = 0.002). The relationship is illustrated in Figure 3, where a linear fit (Pearson r = 0.37, *p* = 0.008) is shown for visualization purposes. However, substantial variability in SE values was observed within individual MOAA/S categories, with values ranging from 64 to 86 within the MOAA/S 2 category. This indicates that similar MOAA/S scores were associated with a wide range of SE values. Correlations between SE and MOAA/S were also examined at later timepoints but did not reach statistical significance, likely reflecting the limited range of MOAA/S scores at deeper sedation levels and the progressive recovery of SE values during the latter part of the procedure.

### 3.4. Sedation Adequacy: Time-in-Zone Analysis

**Figure 4:**
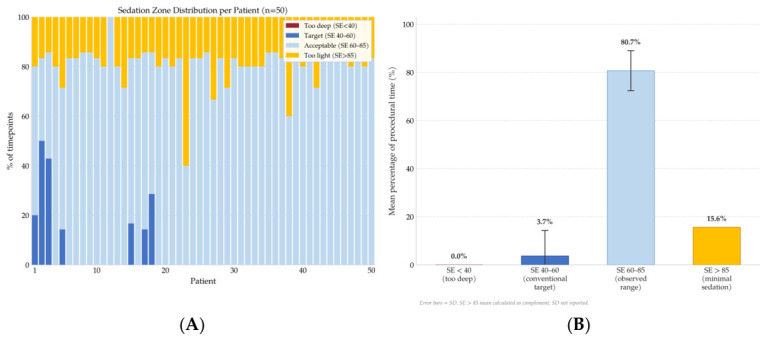
Sedation adequacy analysis based on time-in-zone distribution of State Entropy (SE). (**A**) Individual patient stacked bar chart showing the proportion of recorded timepoints within predefined SE ranges: <40 (too deep), 40–60 (conventional target), 60–85 (acceptable sedation range), and >85 (minimal sedation). (**B**) Group-level summary showing the mean percentage of procedural time spent within each SE zone across all 50 patients. Error bars represent standard deviation where available. The SE > 85 mean was calculated as the complement of the remaining zones; standard deviation is not reported for this category.

The mean SE nadir was 67.2 ± 5.3. As illustrated in Figure 4, the individual patient data (panel A) confirm that the majority of patients spent little or no time within the SE 40–60 interval, while the group-level summary (panel B) demonstrates the predominance of SE 60–85 across the cohort (Table 3).

An exploratory analysis of SE instability in relation to adverse events is presented in Supplementary Table S1; differences did not reach statistical significance, and these results should be considered hypothesis-generating given the limited number of events.

### 3.5. Association Between Patient Characteristics and Entropy Values

**Figure 5:**
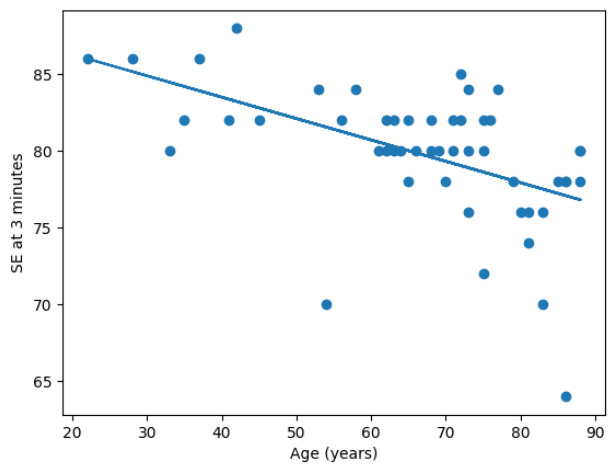
Relationship between age and State Entropy (SE) at 3 min. Scatter plot showing the negative association between age and SE at 3 min (Spearman rho = −0.612, *p* < 0.001). The line represents the linear fit shown for visualization purposes.

The relationship between Entropy values and patient characteristics was examined across intra-procedural timepoints (Table 4 and Figure 5). Age showed a strong negative correlation with SE at 3 min (Spearman rho = −0.612, *p* < 0.001), consistent with deeper early EEG suppression in older patients, which may reflect increased pharmacodynamic sensitivity, age-related changes in spectral substrate, or both. This association was also observed for RE at 3 min (rho = −0.455, *p* = 0.001) and persisted at 5 min for both SE and RE (rho = −0.346 and −0.309, respectively; both *p* < 0.05). No significant correlation between age and SE was observed at baseline or at 10 min.

ASA score showed a different temporal pattern. It was not associated with early Entropy values but demonstrated significant negative correlations at 15 min for both SE (rho = −0.299, *p* = 0.035) and RE (rho = −0.354, *p* = 0.012). Full correlation results are presented in Table 4.

### 3.6. Entropy and Post-Procedural Recovery

The relationship between intraprocedural Entropy values and post-procedural recovery, assessed using Aldrete scores, was examined. Most correlations between Entropy values and Aldrete scores were weak and not statistically significant. Two isolated significant correlations were observed: SE at baseline with Aldrete at 15 min (rho = 0.292, *p* = 0.039), and RE at 3 min with Aldrete at 5 min (rho = 0.318, *p* = 0.024). No significant association was identified between Entropy nadir values and Aldrete scores at 15 min (SE rho = −0.175, *p* = 0.223; RE rho = −0.273, *p* = 0.055). Aldrete scores at 15 min were predominantly high, with most patients achieving values of 9 or 10. The discriminative performance of Entropy and MOAA/S variables for predicting anaesthetic adverse events was evaluated using receiver operating characteristic analysis. AUC values for all evaluated parameters—including MOAA/S minimum (AUC = 0.461), MOAA/S at 3 min (AUC = 0.471), SE at 3 min (AUC = 0.510), and SE nadir (AUC = 0.411)—were close to 0.5, indicating poor and clinically non-significant discriminative ability (Figure S1). This finding is consistent with the limited statistical power of the adverse event analyses and underscores that the observed SE distribution in this cohort constitutes a descriptive characterisation rather than a validated predictive or prescriptive target range.

## 4. Discussion

Before discussing specific findings, several contextual considerations should be noted. This study was conducted in a single centre in a cohort of predominantly elderly, high-ASA patients with a high proportion of malignant diagnoses. The observed SE trajectories and the observed predominance of values within SE 60–85 reflect this specific population and procedural context. Caution is warranted in generalising these findings to younger or lower-risk patients, or to less stimulating endoscopic procedures.

### 4.1. Principal Findings

This prospective observational study characterized SE and RE trajectories during propofol-based sedation for ERCP in a cohort of 50 patients. The main finding was that procedural sedation operated predominantly at SE values between 60 and 85, above the conventional target range of 40–60 derived from general anaesthesia monitoring. Several secondary observations provided preliminary support for the potential relevance of continuous entropy monitoring in this setting: a moderate correlation was identified between SE and MOAA/S at the early timepoints, a pronounced age-related effect on early sedation depth, and a trend toward higher SE variability in patients with adverse events.

### 4.2. Entropy Trajectories and the Adequacy of Conventional Target Ranges

The characteristic U-shaped SE trajectory observed in our cohort—with rapid decline following induction, a nadir near 10 min, and progressive recovery thereafter—reflects the pharmacodynamic profile of propofol during short procedures [7]. It should be noted that the progressive increase in SE values at 20 and 25 min was based on a decreasing number of patients (n = 35 and n = 15 respectively), as shorter procedures had concluded by these timepoints. The apparent late-procedure SE recovery may therefore partly reflect a selection effect, with longer and potentially more complex procedures contributing disproportionately to these timepoints. The mean SE nadir of 67.2 ± 5.3 remained well above the lower bound of the conventionally recommended SE 40–60 window, and no patient reached SE values below 40 at any timepoint. This pattern contrasts with what would be expected during general anaesthesia, where sustained SE values in the 40–60 range are regarded as indicative of an adequate hypnotic state [8].

The observation that patients spent a mean of only 3.7% of procedural time within the SE 40–60 zone, compared with 80.7% within SE 60–85, suggests that the conventional SE 40–60 target range, derived from general anaesthesia, may not reflect the sedation plane typically observed during ERCP in this cohort. The 40–60 range has been established in the context of general anaesthesia, where the aim is suppression of consciousness and prevention of awareness. In contrast, procedural sedation for ERCP targets a different physiological state, characterized by reduced responsiveness with preservation of spontaneous ventilation and airway reflexes, where the level of cortical suppression required may be systematically lower. This interpretation is supported by previous observations that EEG-derived indices during gastrointestinal endoscopy often correspond to higher BIS or entropy values than those associated with general anaesthesia [2] and with published data suggesting that clinically effective sedation for gastrointestinal endoscopy frequently corresponds to BIS or entropy values above those associated with unconsciousness [6,9,10].

The high proportion of patients with at least one SE > 85 timepoint (98%) is largely attributable to the pre-induction baseline and recovery phase, during which propofol effect was minimal; this finding does not indicate inadequate sedation during active procedural phases.

Whether a procedure-specific entropy reference range exists for ERCP, and whether it differs meaningfully from general anaesthesia targets, requires prospective outcome-anchored investigation. Importantly, the absence of meaningful discriminative performance for adverse events across all entropy and clinical variables (AUC values approximately 0.5) confirms that the observed SE 60–85 distribution represents a descriptive characterisation of sedation depth in this cohort, and cannot be interpreted as a validated target range without prospective validation. To our knowledge, this is the first study to characterise SE and RE trajectories using time-in-zone analysis specifically during propofol sedation for ERCP, addressing a gap not covered by prior BIS-focused studies in gastrointestinal endoscopy.

Future validation studies should incorporate predefined candidate outcome anchors, such as patient movement requiring intervention, endoscopist-rated procedural conditions, the occurrence of respiratory adverse events, or post-procedural cognitive assessments, to establish whether entropy-guided sedation within a procedure-specific range translates into measurable clinical benefit.

### 4.3. Relationship Between Entropy and Clinical Sedation Assessment

The moderate positive correlation between SE and MOAA/S at 3 min (Spearman rho = 0.430, *p* = 0.002) indicates a relationship between EEG-derived and observational measures of sedation at an early intra-procedural timepoint. Higher SE values were associated with lighter clinical sedation, consistent with the expected relationship between cortical EEG suppression and responsiveness. However, the correlation was modest, and substantial variability in SE values was observed within individual MOAA/S categories, with values ranging from 64 to 86 within the MOAA/S 2 category. This finding is consistent with the complementary nature of continuous entropy monitoring and clinical sedation assessment, particularly at MOAA/S score levels where further behavioural discrimination becomes limited. This is consistent with previous studies showing that EEG-derived indices correlate with clinical sedation scales but provide greater resolution than intermittent behavioral assessment [11].

The absence of significant correlations between SE and MOAA/S at later timepoints may reflect the limited range of MOAA/S scores at deeper levels of sedation, as well as changes in SE during the recovery phase of the procedure, leading to reduced alignment between the two measures [1]. This interpretation is consistent with the known limitations of ordinal clinical sedation scales in distinguishing between deeper levels of sedation.

### 4.4. Age as a Determinant of Sedation Depth

The strong negative correlation between age and SE at 3 min (rho = −0.612, *p* < 0.001) indicates that older patients experienced deeper early sedation following propofol administration. This pattern was also observed for RE at 3 min and persisted at 5 min, before attenuating at later timepoints. A significant negative correlation between age and RE at baseline (rho = −0.422, *p* = 0.002) was also observed prior to drug administration. This may reflect age-related differences in resting frontal electromyographic activity captured by RE, which incorporates higher-frequency EMG components; however, this finding was not anticipated and should be interpreted cautiously. These findings are consistent with known age-related changes in propofol pharmacokinetics and pharmacodynamics, including reduced clearance and increased sensitivity to its hypnotic effects [12]. The results suggest that older patients may be more susceptible to deeper early sedation at standard induction doses. Whether continuous entropy monitoring could assist in identifying and responding to this effect in clinical practice requires prospective evaluation in interventional study designs where entropy values actively guide sedation titration. These findings are further contextualised by Huang et al. [13], who recently characterised PSI-based sedation monitoring in elderly ERCP patients and similarly identified age as a significant determinant of sedation depth, with older patients demonstrating lower PSI values at equivalent propofol doses. These parallel findings across two spectrally derived indices—PSI and Entropy—that share computational foundations in EEG spectral analysis, strengthen the hypothesis that age-related changes in frontal EEG dynamics represent a consistent and clinically meaningful phenomenon in this population, independent of the specific monitoring algorithm employed.

Notably, ASA score showed a different temporal pattern, with significant correlations emerging at 15 min rather than at early timepoints. This temporal dissociation between age and ASA score effects may reflect the predominance of pharmacokinetic factors—particularly the early peak effect of propofol—in driving age-related variability in sedation depth [7].

### 4.5. SE Variability and Adverse Events

Patients who experienced adverse events, including desaturation, hypotension, and the composite outcome, showed higher within-patient SE variability compared with those who did not. Numerically, the largest difference in SE instability was observed for desaturation events (median 10.2 vs. 7.9 IQR units), though this comparison was based on only 6 events and did not reach statistical significance (*p* = 0.328). The composite adverse event outcome showed the smallest *p*-value (*p* = 0.086), though this too remained non-significant. All comparisons should be interpreted as exploratory given the limited event numbers, and the observed patterns are consistent with the hypothesis that oscillations in sedation depth—reflecting alternating under- and over-sedation—may contribute to haemodynamic and respiratory instability during ERCP [14,15,16,17]. However, these differences did not reach statistical significance in the current study, and the findings should be interpreted as hypothesis-generating. It should be noted that with fewer than 10 events per outcome, these comparisons were substantially underpowered, and the observed trends should be interpreted accordingly. The observed pattern suggests a potential association between greater variability in sedation depth and adverse events, which warrants further investigation in larger studies.

### 4.6. Post-Procedural Recovery and Its Association with Entropy

Post-procedural Aldrete scores were high across the cohort, and most correlations between intraoperative entropy values and recovery scores were weak and not statistically significant. Two isolated associations were observed between SE at baseline and Aldrete at 15 min, and between RE at 3 min and Aldrete at 5 min. The limited variability in Aldrete scores, with most patients achieving values of 9 or 10 at 15 min, likely reduced the ability to detect meaningful associations [18]; notably, psychomotor function remains significantly impaired even when patients achieve an Aldrete discharge score of 10, with average psychomotor recovery at only 60–70% of baseline when Aldrete criteria are met [19], suggesting that this scoring system may not capture more subtle aspects of recovery [20]. Furthermore, the relationship between intraoperative depth-of-sedation monitoring and post-procedural recovery remains incompletely characterized in the endoscopic sedation literature, with current guidelines emphasizing the use of standardized discharge criteria but not specifically addressing correlations between intraoperative EEG parameters and recovery scores [21].

The absence of a significant association between entropy nadir values and Aldrete recovery scores at 15 min is consistent with propofol’s short context-sensitive half-time and rapid redistribution following cessation of infusion [7]. In short-duration ERCP procedures, drug offset is primarily governed by pharmacokinetic rather than pharmacodynamic factors, offering clinical reassurance that transient periods of deeper sedation do not necessarily prolong early recovery, though longer procedures or higher cumulative doses may behave differently. The isolated significant correlations with Aldrete scores and the ROC/AUC analyses should be interpreted with particular caution, as the limited number of events renders these analyses statistically unstable and their clinical significance is uncertain.

### 4.7. Adjunctive Medications

Adjunctive medications were administered to a minority of patients. Midazolam was used in 9 patients (18%), who were slightly younger than the overall cohort (median age 70 versus 72.5 years), consistent with its use for anxiolysis in patients with greater pre-procedural anxiety. Ketamine was administered to 3 patients (6%), all of whom had more than one cannulation attempt and two of whom had angiocholitis, suggesting its use was driven by procedural complexity and analgesic requirements rather than age. Procedure duration in ketamine-treated patients was not longer than the cohort median. Given the small number of patients receiving adjunctive agents and the exploratory nature of the study, formal sensitivity analyses excluding these patients were not performed, as they would further reduce an already limited sample size; however, given the proportions involved, their influence on group-level entropy trajectories is likely limited.

### 4.8. Spectral Substrate and Pharmacological Confounders: Limitations of Numerical Cross-Cohort Comparison

A fundamental limitation of the cross-cohort numerical comparison underlying the time-in-zone analysis warrants explicit acknowledgement. The conventional SE 40–60 target was derived from validation studies in predominantly younger, ASA I–II adults receiving propofol- or sevoflurane-based general anaesthesia [22], where this range corresponds to a specific underlying EEG spectral pattern rather than an absolute numerical threshold. The present cohort differs substantially from this reference population in two respects.

First, aging is associated with progressive reductions in frontal alpha power and increased susceptibility to burst suppression at equivalent propofol concentrations [23,24], directly influencing the computation of spectral entropy indices. The baseline age–RE correlation observed prior to drug administration (rho = −0.422, *p* = 0.002) provides direct empirical support, demonstrating that entropy indices are age-dependent in their measurement properties independent of pharmacological effect.

Second, the multi-drug protocol employed—propofol combined with fentanyl in all patients, midazolam in 18%, and ketamine in 6%—produces a composite EEG signature that differs substantially from propofol monotherapy [24], and it may have contributed to the higher SE values observed. Formal sensitivity analyses comparing entropy trajectories between drug subgroups were not performed, as subgroup sizes preclude meaningful statistical inference.

Taken together, these considerations suggest that the present findings reflect a cohort-specific rather than a universally applicable procedure-specific phenomenon. Future studies should incorporate spectrogram-based monitoring approaches—such as density spectral arrays—to capture the full spectral pattern rather than a single numerical summary, particularly in elderly patients receiving multi-drug sedation regimens [23,24].

### 4.9. Limitations

Several limitations of this study should be acknowledged. First, as a non-randomised, single-center observational study, causal inferences cannot be drawn; however, the primary aim of this study was descriptive—to characterise SE trajectories during ERCP sedation and evaluate their relationship with clinical parameters—for which an observational design is appropriate and sufficient. Second, the sample size of 50 patients, while sufficient for describing entropy trajectories, provides limited statistical power for subgroup analyses and adverse event associations. Third, the study population was predominantly elderly and of high ASA physical status, with a high proportion of malignant diagnoses; the observed SE trajectories and the tentative SE 60–85 reference range may not generalise to younger or lower-risk patients undergoing ERCP, and this should be considered when interpreting the findings.

Fourth, entropy values were monitored continuously but recorded at predefined timepoints, which may not fully capture dynamic changes in sedation depth. Fifth, the SE instability metric used in this study has inherent limitations as a measure of dynamic sedation oscillation. The within-patient range is sensitive to single outlier values and conflates the expected pharmacodynamic decline from awake baseline to nadir with genuine intra-procedural instability. Complementary metrics such as the intra-procedural standard deviation or mean absolute successive difference, restricted to the post-induction phase, would provide a more granular characterisation of sedation depth oscillation and should be pre-specified in future studies. Sixth, sedation management was not protocol-driven and reflected routine clinical practice, introducing variability in propofol dosing and supplementary agent use that may have influenced entropy trajectories. Seventh, operator-level variability in procedural stimulation and technique was not formally analysed, as individual endoscopist data were not recorded. Finally, the SE 60–85 range identified in this study was derived post hoc and should be considered exploratory; prospective studies are required to validate procedure-specific entropy targets for ERCP. Additionally, the time-in-zone analysis was based on SE values recorded at discrete predefined timepoints rather than continuously captured data. Although the CARESCAPE B650 monitor records entropy values continuously and data were additionally stored electronically, the study protocol defined analysis at fixed timepoints without interpolation between measurements. As a result, the calculated proportions of time within each SE zone represent approximations of continuous procedural exposure. The finding that mean time within SE 40–60 was 3.7% should therefore be interpreted as indicative rather than precise, and continuous entropy data analysis in future studies would provide a more accurate characterisation of time-in-zone distributions. Furthermore, the fixed-timepoint sampling protocol did not capture procedural milestone timestamps—such as scope insertion, cannulation, sphincterotomy, and stent placement—precluding alignment of SE trajectories with specific stimulation events. As a result, it is not possible to determine whether the SE nadir observed at 10 min reflects peak propofol effect-site concentration, a low-stimulation phase preceding cannulation, or a combination of both. This limits the procedure-specific interpretability of the trajectory and represents an important direction for future research using event-synchronised EEG recording.

Future research should focus on the prospective validation of procedure-specific entropy target ranges and on evaluating the clinical impact of entropy-guided sedation protocols in ERCP.

## 5. Conclusions

The findings of this exploratory observational study should be interpreted in the context of their descriptive design and hypothesis-generating intent. Continuous entropy monitoring during propofol sedation for ERCP showed that observed sedation levels were predominantly within SE values of 60–85, above the conventional 40–60 range derived from general anesthesia. This finding aligns with prior studies demonstrating that EEG-derived index values during endoscopic sedation are generally higher than those associated with general anaesthesia [9,10].

Age was a key determinant of early sedation depth, with older patients demonstrating greater early EEG suppression, consistent with well-established age-dependent changes in propofol-induced EEG dynamics, including decreased alpha and delta power, lower BIS values at loss of consciousness, and increased susceptibility to burst suppression in elderly patients [13]. Whether the age-related differences observed in this study reflect increased pharmacodynamic sensitivity, age-related changes in spectral substrate, or both factors warrants further investigation.

Taken together, these findings suggest that in elderly, high-ASA patients receiving propofol-based multi-drug sedation for ERCP, numerical SE values differ systematically from conventional general anaesthesia-derived target ranges. Whether this constitutes a true procedure-specific phenomenon or a cohort-specific observation requires prospective outcome-anchored validation. Future interventional studies should incorporate spectrogram-based monitoring approaches and pre-specified outcome anchors—such as patient movement, endoscopist-rated procedural conditions, and respiratory adverse events—to establish whether entropy-guided sedation titration translates into meaningful clinical benefit in this population.

## Figures and Tables

**Table 1:** Patient characteristics.

Variable	Value
Demographics	
Age, years	72.5 (65.0–79.0)
Sex, male	24 (48.0%)
Clinical Status	
ASA score	3.0 (2.0–3.0)
Hypertension	31 (62.0%)
Pulmonary disease	9 (18.0%)
Anticoagulant therapy	11 (22.0%)
Antiplatelet therapy	18 (36.0%)
Statin use	20 (40.0%)
Procedural Characteristics	
Procedure duration, min	22.5 (20.0–30.0)
Cannulation attempts	3.0 (2.0–5.0)
Wirsung cannulations	0.0 (0.0–1.0)
Stent placement	29 (58.0%)
Malignant diagnosis	28 (56.0%)
Sedation and Medication	
Propofol dose, mg	130.0 (105.0–185.0)
Fentanyl dose, µg	100 (100–200)
Midazolam administration	9 (18.0%)
Midazolam dose, mg *	1.0 (1.0–1.0)
Ketamine administration	3 (6.0%)
Ketamine dose, mg *	20.0 (20.0–40.0)
Adverse Events	
Desaturation	6 (12.0%)
Hypotension (any episode)	8 (16.0%)
Bradycardia	5 (10.0%)
Tachycardia	7 (14.0%)
Recovery and Outcomes	
Aldrete score at 15 min	10.0 (9.0–10.0)

* Reported for patients who received the drug only.

**Table 2:** Descriptive SE and RE trajectories over time.

Timepoint	SE—n	SE—Median (IQR)	SE—Mean ± SD	RE—n	RE—Median (IQR)	RE—Mean ± SD
Baseline	50	90.0 (88.0–90.8)	89.3 ± 1.5	50	98.5 (98.0–99.8)	98.4 ± 1.3
3 min	50	80.0 (78.0–82.0)	79.8 ± 4.4	50	88.0 (82.2–92.0)	86.5 ± 5.8
5 min	50	74.0 (70.0–77.5)	73.0 ± 5.2	50	78.0 (74.0–80.0)	77.3 ± 5.6
10 min	50	70.0 (68.0–70.0)	68.8 ± 5.3	50	72.0 (70.0–76.0)	72.8 ± 5.8
15 min	50	71.0 (68.0–76.0)	72.1 ± 7.4	50	76.0 (72.0–82.0)	77.2 ± 8.5
20 min	35	72.0 (69.0–80.0)	74.0 ± 6.9	35	80.0 (73.0–89.0)	80.7 ± 8.9
25 min	15	82.0 (80.5–84.5)	81.8 ± 4.5	15	90.0 (88.5–92.0)	89.1 ± 4.7

**Table 3:** Time-in-zone analysis.

Metric	Value
Patients with any timepoint too deep (SE < 40)	0 (0.0%)
Patients with any timepoint too light (SE > 85)	49 (98.0%)
Mean % time in target zone (SE 40–60)	3.7% (SD 10.6)
Mean % time in acceptable zone (SE 60–85)	80.7% (SD 8.3)
Mean SE nadir	67.2 (SD 5.3)

**Table 4:** Correlation between patient characteristics (age and ASA score) and Entropy values across procedural timepoints.

Predictor	Outcome	Spearman Rho	*p*-Value
Age (years)	SE at 3 min	−0.612	<0.001
Age (years)	RE at 3 min	−0.455	0.001
Age (years)	RE baseline	−0.422	0.002
ASA score	RE at 15 min	−0.354	0.012
Age (years)	SE at 5 min	−0.346	0.014
Age (years)	RE at 5 min	−0.309	0.029
ASA score	SE at 15 min	−0.299	0.035
Age (years)	SE baseline	−0.169	0.241
Age (years)	SE at 15 min	−0.16	0.266
ASA score	SE at 3 min	−0.16	0.267
Age (years)	SE at 10 min	−0.154	0.286
Age (years)	RE at 10 min	−0.076	0.601
ASA score	RE at 3 min	−0.066	0.65
Age (years)	RE at 15 min	−0.063	0.662
ASA score	RE baseline	−0.039	0.787
ASA score	SE at 10 min	−0.039	0.788
ASA score	SE baseline	−0.034	0.814
ASA score	RE at 5 min	−0.033	0.818
ASA score	RE at 10 min	−0.025	0.861
ASA score	SE at 5 min	0.017	0.905

All correlations were computed on the full study sample (n = 50).

## Data Availability

The data presented in this study are available on reasonable request from the corresponding author. The data are not publicly available due to privacy and ethical restrictions.

## Supplementary Materials

The following supporting information can be downloaded at https://www.mdpi.com/article/10.3390/medicina62061047/s1. Figure S1: Receiver operating characteristic (ROC) curves for Entropy and MOAA/S variables as predictors of anaesthetic adverse events; Figure S2: Relationship between Entropy nadir values and post-procedural recovery; Table S1: SE instability in relation to adverse events.

## References

[1] Heavner M.S., Gorman E.F., Linn D.D., Yeung S.Y.A., Miano T.A. (2022). Systematic Review and Meta-analysis of the Correlation between Bispectral Index (BIS) and Clinical Sedation Scales: Toward Defining the Role of BIS in Critically Ill Patients. Pharmacother. J. Hum. Pharmacol. Drug Ther..

[2] Amornyotin S. (2013). Sedation and Monitoring for Gastrointestinal Endoscopy. World J. Gastrointest. Endosc..

[3] Punjasawadwong Y., Phongchiewboon A., Bunchungmongkol N. (2014). Bispectral Index for Improving Anaesthetic Delivery and Postoperative Recovery. Cochrane Database Syst. Rev..

[4] Jones J.H., Nittur V.R., Fleming N., Applegate R.L. (2021). Simultaneous Comparison of Depth of Sedation Performance between SedLine and BIS During General Anesthesia Using Custom Passive Interface Hardware: Study Protocol for a Prospective, Non-Blinded, Non-Randomized Trial. BMC Anesthesiol..

[5] Leslie K., Myles P.S., Forbes A., Chan M.T.V., Short T.G., Swallow S.K. (2005). Recovery from Bispectral Index-Guided Anaesthesia in a Large Randomized Controlled Trial of Patients at High Risk of Awareness. Anaesth. Intensive Care.

[6] Mandel J.E., Tanner J.W., Lichtenstein G.R., Metz D.C., Katzka D.A., Ginsberg G.G., Kochman M.L. (2008). A Randomized, Controlled, Double-Blind Trial of Patient-Controlled Sedation with Propofol/Remifentanil Versus Midazolam/Fentanyl for Colonoscopy. Anesth. Analg..

[7] Sahinovic M.M., Struys M.M.R.F., Absalom A.R. (2018). Clinical Pharmacokinetics and Pharmacodynamics of Propofol. Clin. Pharmacokinet..

[8] Dinu A.R., Rogobete A.F., Popovici S.E., Bedreag O.H., Papurica M., Dumbuleu C.M., Velovan R.R., Toma D., Georgescu C.M., Trache L.I. (2020). Impact of General Anesthesia Guided by State Entropy (SE) and Response Entropy (RE) on Perioperative Stability in Elective Laparoscopic Cholecystectomy Patients—A Prospective Observational Randomized Monocentric Study. Entropy.

[9] Paspatis G.A., Tribonias G., Manolaraki M.M., Konstantinidis K., Chainaki I., Theodoropoulou A., Vardas E., Chlouverakis G. (2011). Deep Sedation Compared with Moderate Sedation in Polyp Detection during Colonoscopy: A Randomized Controlled Trial: Deep vs Moderate Sedation in Colonoscopy. Color. Dis..

[10] Von Delius S., Salletmaier H., Meining A., Wagenpfeil S., Saur D., Bajbouj M., Schneider G., Schmid R., Huber W. (2012). Bispectral Index Monitoring of Midazolam and Propofol Sedation during Endoscopic Retrograde Cholangiopancreatography: A Randomized Clinical Trial (the EndoBIS Study). Endoscopy.

[11] Lim S., Cho K., Lee W., Kim J., Bang J., Ki S. (2022). Comparison of the Performance of Phase Lag Entropy and Bispectral Index for Monitoring the Depth of Sedation under Dexmedetomidine Sedation: A Prospective, Observational, and Non-Inferiority Trial. J. Clin. Anesth..

[12] Kim Y., Park J., Chung W., Jo Y., Oh C., Hong B., Kim S. (2025). Age-Related Electroencephalographic Delta and Alpha Oscillations During Sedation with Target-Controlled Propofol Infusion. J. Clin. Med..

[13] Huang L., Liu L., Lu Y., Zhuang M., Dou W., Liu H., Ji F., Peng K. (2025). Assessing Sedation Depth with PSI in Elderly ERCP Patients: A Prospective Cohort Study. Clin. Interv. Aging.

[14] Krauss B., Green S.M. (2006). Procedural Sedation and Analgesia in Children. Lancet.

[15] Wall B.F., Magee K., Campbell S.G., Zed P.J. (2017). Capnography versus Standard Monitoring for Emergency Department Procedural Sedation and Analgesia. Cochrane Database Syst. Rev..

[16] Schick A., Driver B., Moore J.C., Fagerstrom E., Miner J.R. (2019). Randomized Clinical Trial Comparing Procedural Amnesia and Respiratory Depression Between Moderate and Deep Sedation with Propofol in the Emergency Department. Acad. Emerg. Med..

[17] Mathews D.M., Clark L., Johansen J., Matute E., Seshagiri C.V. (2012). Increases in Electroencephalogram and Electromyogram Variability Are Associated with an Increased Incidence of Intraoperative Somatic Response. Anesth. Analg..

[18] McGrath B., Chung F. (2003). Postoperative Recovery and Discharge. Anesthesiol. Clin. N. Am..

[19] Cohen L.B., DeLegge M.H., Aisenberg J., Brill J.V., Inadomi J.M., Kochman M.L., Piorkowski J.D. (2007). AGA Institute Review of Endoscopic Sedation. Gastroenterology.

[20] Willey J., Vargo J.J., Connor J.T., Dumot J.A., Conwell D.L., Zuccaro G. (2002). Quantitative Assessment of Psychomotor Recovery after Sedation and Analgesia for Outpatient EGD. Gastrointest. Endosc..

[21] Early D.S., Lightdale J.R., Vargo J.J., Acosta R.D., Chandrasekhara V., Chathadi K.V., Evans J.A., Fisher D.A., Fonkalsrud L., Hwang J.H. (2018). Guidelines for Sedation and Anesthesia in GI Endoscopy. Gastrointest. Endosc..

[22] Viertiö-Oja H., Maja V., Särkelä M., Talja P., Tenkanen N., Tolvanen-Laakso H., Paloheimo M., Vakkuri A., Yli-Hankala A., Meriläinen P. (2004). Description of the Entropy^TM^ Algorithm as Applied in the Datex-Ohmeda S/5^TM^ Entropy Module. Acta Anaesthesiol. Scand..

[23] Purdon P.L., Pavone K.J., Akeju O., Smith A.C., Sampson A.L., Lee J., Zhou D.W., Solt K., Brown E.N. (2015). The Ageing Brain: Age-Dependent Changes in the Electroencephalogram during Propofol and Sevoflurane General Anaesthesia. Br. J. Anaesth..

[24] Akeju O., Brown E.N. (2017). Neural Oscillations Demonstrate That General Anesthesia and Sedative States Are Neurophysiologically Distinct from Sleep. Curr. Opin. Neurobiol..

