# Supplementary material for: EEG-Derived Entropy Monitoring During Propofol Sedation for ERCP: Sedation Profiles, Age-Related Effects, and Implications for Procedure-Specific Target Ranges
Source: Medicina (Kaunas). 2026 May 28;62(6):1047. doi: 10.3390/medicina62061047 (PMC13304176; doi:10.3390/medicina62061047)
Supplement: Supplementary file 1 [file medicina-62-01047-s001.zip › medicina-4310531-supplementary.pdf]

medicina

MDPI

Supplementary Materials

The following supplementary figures and tables are available:

- Figure S1. Receiver operating characteristic (ROC) curves for Entropy and MOAA/S variables as predictors of anaesthetic adverse events.
- Figure S2. Relationship between Entropy nadir values and post-procedural recovery.
- Table S1. SE instability in relation to adverse events

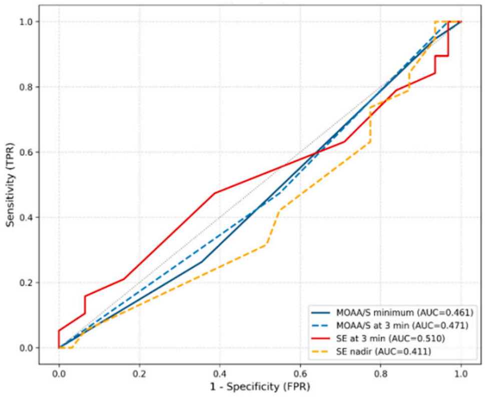

Figure S1. Receiver operating characteristic (ROC) curves for Entropy and MOAA/S variables as predictors of anaesthetic adverse events. Receiver operating characteristic (ROC) curves evaluating the discriminative performance of selected variables for predicting anaesthetic adverse events in the Entropy subgroup (n = 50). Variables included MOAA/S minimum, MOAA/S at 3 minutes, SE at 3 minutes, and SE nadir. All variables demonstrated limited discriminative ability, with area under the curve (AUC) values close to 0.5,

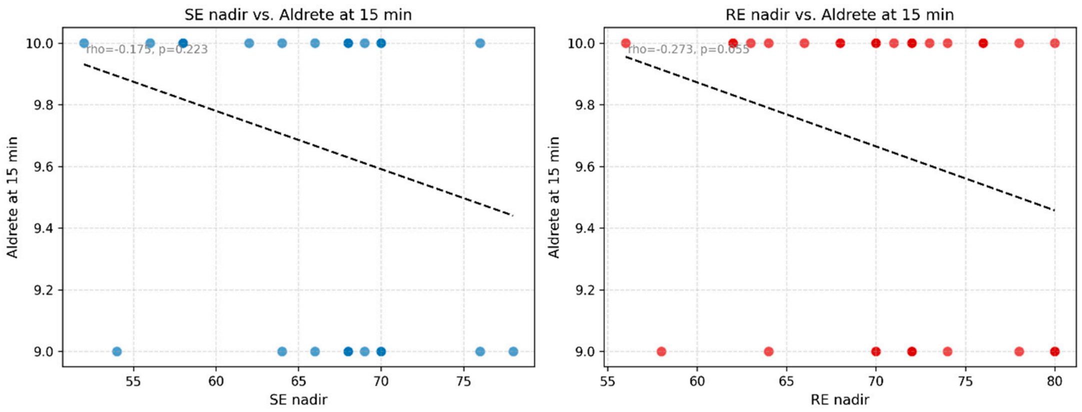

indicating poor predictive performance. The full study cohort of 50 patients was used for this analysis.

Figure S2. Relationship between Entropy nadir values and post-procedural recovery
Scatter plots illustrate the relationship between State Entropy (SE) nadir (left) and Response Entropy (RE) nadir (right) with Aldrete score at 15 minutes. No significant correlations were observed (SE: rho = −0.175, p = 0.223; RE: rho = −0.273, p = 0.055), indicating no meaningful association between intra-procedural Entropy nadir values and early recovery. The full study cohort of 50 patients was used for this analysis.

medicina MDPI

Table S1. SE instability in relation to adverse events

Outcome	Adverse Event (+) Median instability (IQR)	Adverse Event (−) Median instability (IQR)	p-value
Composite adverse event	8.4 (7.8–9.4)	7.4 (6.9–8.9)	0.086
Desaturation	10.2 (9.0–11.4)	7.9 (7.1–9.1)	0.328
Hypotension	8.6 (7.7–10.3)	7.8 (7.1–9.0)	0.367

Between-group differences assessed by Mann–Whitney U test. SE instability defined as the difference between maximum and minimum SE values recorded during the procedure. Given the limited number of events per outcome (range 6–8), these comparisons were substantially underpowered and should be considered hypothesis-generating only.
